# Even Four Minutes of Poor Quality of CPR Compromises Outcome in a Porcine Model of Prolonged Cardiac Arrest

**DOI:** 10.1155/2013/171862

**Published:** 2013-12-02

**Authors:** Heng Li, Lei Zhang, Zhengfei Yang, Zitong Huang, Bihua Chen, Yongqin Li, Tao Yu

**Affiliations:** ^1^Emergency Department, Sun Yat-Sen Memorial Hospital of Sun Yat-Sen University, Guangzhou 510120, China; ^2^Institute of Cardiopulmonary Cerebral Resuscitation, Sun Yat-Sen University, Guangzhou 510120, China; ^3^Department of Emergency Medicine, Taiping People's Hospital, Medical School of Jinan University, Dongguan 523905, China; ^4^Emergency Department, Southwest Hospital of the Third Military Medical University, Chongqing 400038, China; ^5^School of Biomedical Engineering, Third Military Medical University and Chongqing University, Chongqing 400038, China

## Abstract

*Objective*. Untrained bystanders usually delivered suboptimal chest compression to victims who suffered from cardiac arrest in out-of-hospital settings. We therefore investigated the hemodynamics and resuscitation outcome of initial suboptimal quality of chest compressions compared to the optimal ones in a porcine model of cardiac arrest. *Methods*. Fourteen Yorkshire pigs weighted 30 ± 2 kg were randomized into good and poor cardiopulmonary resuscitation (CPR) groups. Ventricular fibrillation was electrically induced and untreated for 6 mins. In good CPR group, animals received high quality manual chest compressions according to the Guidelines (25% of animal's anterior-posterior thoracic diameter) during first two minutes of CPR compared with poor (70% of the optimal depth) compressions. After that, a 120-J biphasic shock was delivered. If the animal did not acquire return of spontaneous circulation, another 2 mins of CPR and shock followed. Four minutes later, both groups received optimal CPR until total 10 mins of CPR has been finished. *Results*. All seven animals in good CPR group were resuscitated compared with only two in poor CPR group (*P* < 0.05). The delayed optimal compressions which followed 4 mins of suboptimal compressions failed to increase the lower coronary perfusion pressure of five non-survival animals in poor CPR group. *Conclusions*. In a porcine model of prolonged cardiac arrest, even four minutes of initial poor quality of CPR compromises the hemodynamics and survival outcome.

## 1. Introduction

Cardiac arrest (CA) is still a major public health problem around the world. It might contribute to more than 800,000 victims in western industrialized society and 540,000 in developing China annually with limited survival rate [1–3]. Over the decades, the implementation of survival chain has obtained beneficial outcomes from out-of-hospital cardiac arrest (OHCA) in some communities. Therefore, it is generally accepted and undoubtfully regarded that the measures of early chest compression and rapid defibrillation were the cornerstone of effective resuscitation especially in the absence of EMS personnel in out-of-hospital setting.

Although scene rapid defibrillation had been feasible with the aid of automatic external defibrillator (AED) and public access defibrillation (PAD), the quality of chest compression is still a critical determinant in preshock interval. Based on investigation data on animal and human, sufficient blood flow of vital organs produced by optimal cardiopulmonary resuscitation (CPR) was supposed to hold promise to the successful defibrillation following ventricular fibrillation (VF) and survival discharged with intact neurological behavior [4–6]. However, most CA patients can not usually received CPR or effective CPR whether witnessed or not. It was reported that this percentage was 37 to 42 in OHCA for those who received bystander CPR in which only 28% adhered to the target depth in the first 5 mins of CPR as guideline recommended [7, 8].

The data stated that shallow compression depth, inappropriate rate, incomplete thoracic recoil, and unnecessary compression interruption usually lead to the failure on establishment of spontaneous circulation [1, 9–11]. All these deficiencies that exacerbated outcome were commonly seen and inevasible in actual resuscitation episode, especially for those laypersons without basic life support training. In other words, bystander CPR improves survival in CA [12], however, the quality of bystander CPR should be monitored and focused [13, 14]. In the present study, we therefore sought to evaluate the hemodynamics and resuscitation outcome in those received suboptimal quality of CPR in initial four minutes compared to good CPR originally. We hypothesized that the initial suboptimal CPR might compromise the resuscitation outcomes of cardiac arrest animals.

## 2. Method

### 2.1. Study Design

This prospective, randomized, single center and controlled experiment was designed to simulate the suboptimal bystander CPR and investigate its consequence. Experiments were performed in an established swine model of electrically induced cardiac arrest in Laboratory Animal Center of Sun Yat-sen University (Guangzhou, China). All animals received humane care and the experiments were conducted after approval of the Animal Ethics Committee, Sun Yat-sen University. The protocol was performed according to institutional guidelines.

### 2.2. Animal Preparation

Fourteen male Yorkshire pigs, weighting 30 ± 2 kg, were fasted overnight except for free excess to water. Anesthesia was initiated by intramuscular injection of ketamine (20 mg/kg) and completed by ear vein injection of sodium pentobarbital (30 mg/kg). Additional doses of sodium pentobarbital (8 mg/kg) were injected at intervals of approximately 1 hr to maintain anesthesia. A cuffed endotracheal tube was advanced into the trachea. Animals were mechanically ventilated with a volume-controlled ventilator (T-Bird AVIII, Bird Products Corporation, Palm Springs, CA), with a tidal volume of 15 mL/kg and FiO_2_ of 21%.

For the measurement of aortic pressure, a 6F fluid-filled angiographic catheter (model 070, Cordis Corporation, Miami Lakes, FL, USA) was advanced from the surgically exposed right femoral artery into the thoracic aorta. For measurements of right atrial pressure and pulmonary arterial pressure, a 7F pentalumen thermodilution-tipped catheter (model 131HF7, Swan-Ganz TD, Edwards Life sciences, CA, USA) was advanced from the surgically exposed right femoral vein and flow directed into the pulmonary artery. For inducing VF, a 5-Fr pacing catheter (Cordis Corporation, Miami Lakes, FL, USA) was advanced from the right jugular vein into the right ventricle until an endocardial electrocardiogram confirmed endocardial contact via a multi parameter monitor (78352C, HP Corporation, Palo Alto, CA, USA). The hard gel type of adult defibrillation/pacing pads (stat-padz, Zoll Medical Corporation, Chelmsford, MA, USA) was applied with an anterior to lateral placement. An accelerometer-based handheld CPR device (CPR-D-padz, Zoll Medical Corporation, Chelmsford, MA, USA) was placed on the surface of the animal's chest just above the heart and underneath the rescuer's hands during chest compression. Cardiac output was measured by the thermodilution technique with the aid of a cardiac output computer (Baxter COM-2TM, Edwards Division, Santa Ana, CA, USA) after a bolus injection into the right atrium of 5 mL cold saline solution, which had been maintained at a temperature between 0°C and 2°C. Aortic blood gases were measured with the aid of a handheld blood analyzer (model CG4+ Cartridge, Abbott i-STAT System, Princeton, NJ, USA). Respiratory frequency was adjusted to maintain PetCO_2_ between 35 mmHg and 40 mmHg before inducing cardiac arrest and when mechanical ventilation was resumed after resuscitation.

### 2.3. Experimental Procedure

After collection of baseline data, cardiac arrest was induced with a 2 mA alternating current delivered to the endocardium of the right ventricle. After VF had been successfully induced, mechanical ventilation was discontinued and cardiac arrest was untreated for a total of 6 mins. Animals were then randomized to one of the following two groups: good CPR, where manual chest compression was performed by an emergency medical doctor at a rate of 100 per min and a depth comparable to 25% of the anterior posterior diameter of the chest, which represented approximately 50 mm; poor CPR, where chest compression was operated by another emergency medical doctor at the same rate, but the chest was compressed to 70% of the depth of good CPR group, which was equivalent to approximately 17% of the anterior posterior diameter of 35 mm [8, 15]. The poor depth represented a value corresponding to the average suboptimal depth of compression recorded during out-of-hospital CPR [8, 16, 17]. During chest compression, the rescuer was blinded from the monitored compression depth and CPP values but with acknowledgment of whether his compressions were below or above 38 mm. The animal's chest wall was allowed to completely recoil in both groups. The animals were manually ventilated with a bag-valve device during CPR. Chest compression was synchronized to provide a compression/ventilation ratio of 30 : 2 with equal compression-relaxation intervals. No epinephrine or other vasopressor agents were administered. After 2 mins of compression in each group, a defibrillation was attempted with a single 120-J rectilinear biphasic shock (M-Series, Zoll Medical corporation, Chelmsford, MA, USA). Chest compression was immediately resumed followed by ECG rhythm analysis within 5 secs until confirmation of spontaneous circulation. The defibrillation attempt was regarded as successful when the electrical shock converted VF to an organized rhythm with a mean aortic pressure of ≥60 mmHg for an interval ≥10 sec [17]. If spontaneous circulation was not restored, in good CPR group, high quality chest compressions were continued for another 2 mins, after which defibrillation was attempted with another single 120 J shock, this sequence was repeated for a maximum of 5 cycles. But in poor CPR group, another 2 mins of low quality chest compressions were continued, followed another single 120 J shock, and then high quality of CPR immediately followed after defibrillation until the spontaneous circulation was restored. Otherwise, resuscitation procedures were terminated after a maximum of another 3 high quality CPR cycles.

Catheters were removed after 1 hr of postresuscitation monitoring, and the animals were euthanized by injection of 150 mg/kg intravenous pentobarbital.

### 2.4. Measurement

Baseline measurements were obtained, including ECG, the aortic pressure, right atrial pressure, cardiac output, and blood gas analysis. The ECG, pressure measurements and acceleration signals were continuously measured and recorded through a data acquisition system supported by Windaq hardware/software (Dataq Instruments Inc., Akron, OH, USA) at a sample rate of 300 Hz. The coronary perfusion pressure (CPP) was digitally computed from the differences in time-coincident diastolic aortic and right atrial pressures. The compression rate and depth were calculated from the double integration of acceleration signals recorded from accelerometer by MATLAB7.0 (The Math Works, Inc., Natick, MA, USA).

### 2.5. Statistical Analyses

Data are presented as mean ± standard deviation (SD). Differences in compression depth and CPP between the two groups were analyzed by two-tailed Student's *t*-test for independent samples test. A two-tailed Fisher's exact test was performed for rate comparison. A *P* value <0.05 was regarded as statistically significant.

## 3. Results

Baseline measurements did not differ significantly between the two groups before inducing cardiac arrest (Table 1).

**Figure 1:**
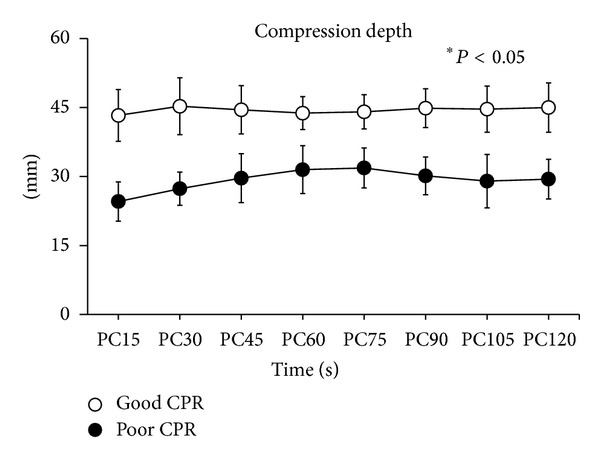
Comparison of compression depth values between the two groups during initial 2 mins of cardiopulmonary resuscitation. *P < 0.05. PC = chest compression.

**Figure 2:**
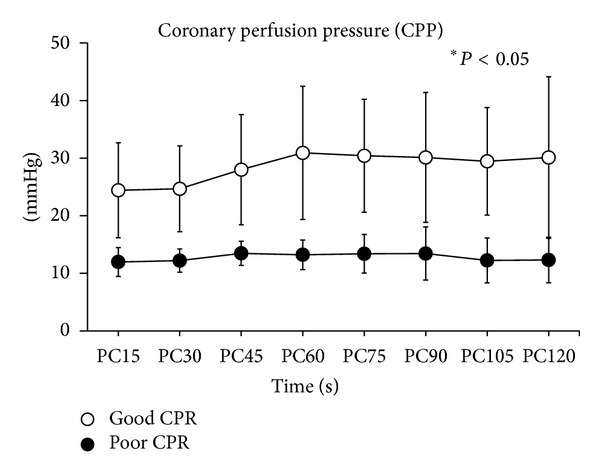
Comparison of coronary perfusion pressure (CPP) values between the two groups during initial 2 mins of cardiopulmonary resuscitation. *P < 0.05. PC = chest compression.

During initial 2 mins of CPR, the measured compression depth was ranged from 19.00 to 38.50 mm in poor CPR group and between 35.20 and 57.00 mm in good CPR group. As shown in Figure 1, the compression depth was significantly higher in good CPR group during the first 2 mins of chest compression (*P* < 0.05). As anticipated, CPP was significantly higher in good CPR group compared with poor CPR group (*P* < 0.05, Figure 2).

**Figure 3:**
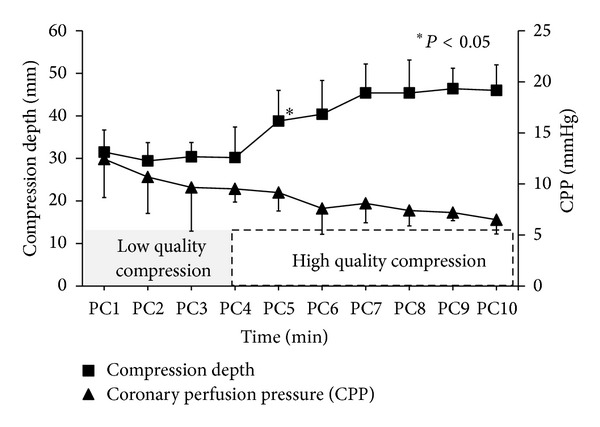
The characteristics of compression depth and coronary perfusion pressure (CPP) in poor CPR group during the entire 10 mins of cardiopulmonary resuscitation procedure. PC = chest compression.

In poor CPR group, the measured compression depth of the first 4 mins of CPR significantly increased after is being changed to good quality compression for the last 6 mins of CPR (30.40 ± 4.70 versus 44.70 ± 6.80, *P* < 0.05). However, the CPP of the animals in this group was not significantly increased correspondingly, as shown in Figure 3.

The defibrillation success rate for the first shock was higher in the good CPR group than in the poor CPR group, but a statistical significance was not achieved (100% versus 71.43%, *P* = 0.46). In poor CPR group, although VF was terminated in 5 pigs after the first shock, 3 animals were sustained in pulseless electric activity (PEA) without ROSC after 10 mins of resuscitation efforts.

All of the 7 animals had ROSC after high quality compressions, while only 2 of the animals had ROSC with 4 mins of low quality compressions (100% versus 28.57%, *P* = 0.021). No rib fractures were observed in both groups.

## 4. Discussion

Our present study demonstrated that initial 4 mins of low quality compression followed by high quality of CPR compromised the outcomes significantly compared with good CPR from the beginning. Additionally, we also found that coronary flow produced by subsequent optimal chest compression could not provide a favorable outcome to those who experienced a low quality of CPR.

Base on previous studies and the current guideline, early and immediate bystander CPR was of importance in treating arrest patients before paramedic arrived, and if it is available, it may improve outcome on survival and neurological function. However, initiation of CPR for a bystander was still hesitating and the quality of this CPR was rarely satisfying. In a perspective observational trial by Kitamura and his team [7], they pointed out there were only 40% laypersons that which tended to perform CPR when witnessed a collapse patient. In the scenarios of cardiac arrest, only 24% of chest compressions performed by untrained laypersons reached the target depth of 38 to 51 mm. The situation still did not take a favorable turn when bystander was a professional physician. Studies carried out by Wik and his colleagues [8] had demonstrated that only 30% of compression depth reached a target value of 31–50 mm in the first 5 mins of CPR and even undertook by ambulance personnel, and nearly half of those compressions (47%) did not achieve the adequate depth even under the condition of application of automated feedback system to assist CPR. Incomplete compression was usually performed in prehospital setting either by layperson or physician.

For chest compression, the fact that CPP was a positive associated with compression depth had been well documented. Sufficient compression depth may bring better blood perfusion to cardiomyodium and produce optimistic resuscitation outcome in animal model of prolonged VF and CPP of ≥15 mmHg in the period of chest compression was considered as an essential condition with the purpose of subsequently successful electrical shock and return of spontaneous circulation [18]. In our present study, it maintained a level of 12 to 15 mmHg in poor CPR comparing with 25 to 30 mmHg in good CPR during the first 2 minutes of chest compression. Similarly, Babbs et al. firstly demonstrated a linear relationship displayed between depth and cardiac output in the range of 23 to 60 mm in the canine model of VF [19]. Besides, according to analyzing the electrocardiogram waveform of VF during compression, Li and his colleagues also concluded that CPP had improved accompanying with the increasing depth [17]. However, in our present study, when compression was transformed from suboptimal to optimal pattern, CPP still persistently declined even a 6 mins of optimal compression was provided. One of the possible explanations this decreasing CPP may be presented with an elevated right atrial pressure contributed to a “stone heart” observed in final autopsy and described by Ventura-Clapier as global ischemic contracture resulting in firm myocardium [20]. It was a deleterious network that decreased coronary blood flow exacerbating ischemia-induced myocardial stiffness when spontaneously coupled with the gradual rising right atrial pressure further precluded coronary perfusion.

The other explanation of deteriorative CPP in following optimal compression might be partially contributed to the decreasing compliance of chest. After 4 mins of low quality of compression, the thoracic elasticity decreased. Then incomplete recoil of chest wall and subsequently decreased CPP and myocardial blood flow even only 10–20% leaning attended in CPR.

Rapid defibrillation has been recommended as a critical and primary treatment for cardiac arrest with initial shockable rhythm as VF or pulseless ventricular tachycardia (VT) [21]. To produce higher success of defibrillation, outcome was primarily determined by two factors: shock time and blood flow of myocardium. In our present study, shock was attempted every 2 mins when ECG was still VF or VT. It was also coincident with the current guideline as 5 cycles of CPR (approximately 2 mins) following by a single 120-J shock. The blood perfusion of heart was essentially associated with performance of CPR. Delayed shocks usually indicated prolonged ischemia and poor CPR brought insufficient perfusion to stiff myocardium. In an observational study of adult cardiac resuscitation [22], the investigator demonstrated that successful defibrillation was associated with shorter preshock pause and higher mean compression depth. Similar results came from a laboratory investigation, the investigator concluded that coronary flow had a strong positive relationship with CPP and the final resuscitation depended on this “threshold CPP” [23]. This might be the answer that two animals in poor CPR group achieved ROSC in first shock with average CPP of 13.50 to 13.80 mmHg, which was close to 15 mmHg.

The finding of this study indicated that there was no statistic difference of the first shock success in both groups. However, 5 animals in poor CPR group failed to return perfused rhythm and functional arterial pressure which finally lead to the significant difference with subsequent final ROSC. For a cardiac arrest porcine model, 6 mins of untreated VF was not long enough to guarantee the difference. A canine model of 5 mins of VF demonstrated that immediate defibrillation without preshock CPR brought none of animals ROSC (0/10), but resulted in 30% successful defibrillation (3/10) [24]. In a prospective cohort study, Stiell found that there was only of 25.7% patients who returned spontaneous circulation with 36.6% of bystander CPR and 46% of EMS compression depth within recommended range. As previously reported, they also did not notify the outcome in those bystander and subsequent EMS CPR group but declared a strong association between survival outcomes and increased compression depth [25]. In a porcine model of 4 mins of VF, Wu compared two different patterns of chest compression and found that the standard compression (rate: 100 ± 5 cpm, depth: 50 ± 1 mm) produced higher ROSC and survival rate than that in simulated clinical compression (rate: 80 ± 5 cpm; depth: 37 ± 1 mm) [26]. Besides, they acquired similar results as ours in shock attempts without consideration of resuscitation procedure. These conclusions may be partially supported by the concept of “circulatory phase,” a time-duration definition that ranges from approximately 4 to 10 mins of VF [27]. Outcomes were prone to be improved when some limited blood circulation with partial substrates was established prior to defibrillation. In our present study, it was the 2 mins of optimal CPR rather than 4 mins of suboptimal CPR, which could make the different ischemic myocardial condition to prepare for the coming counter shock.

It is well known that, with every minute without CPR following sudden cardiac arrest, the probability of survival reduces by 7%–10% per minute [28]. When bystander CPR is delivered, the patient stands a better chance as the probability for survival reduces to 3%-4% per minute. Overall, bystander CPR increases that survival 2-3 times compared to no bystander CPR [13, 14, 29]. When Health Care Professionals deliver quality CPR, research indicates survival rates can increase 4 times, compared to poor CPR [30–32]. Our present study also demonstrated the importance of CPR quality in the initial 4 mins during CPR. In most regions and countries, it can be speculated that no less than 4 mins would be taken to activate and receive EMS assist without satisfying communication and traffic condition [33]. Instead of health care professionals, bystander CPR was encouraged to deliver these basic life support as soon as witnessed an arrest presumed a cardiac origin. The initial quality of compression should be guaranteed by the rescuer, so good training system and useful tools for CPR quality monitor and guidance were totally welcome for the future implementation [34].

We realized that there were some limitations in this study. Firstly, we did not compare the poor CPR group with prolonged (10 mins) untreated VF animals to evaluate if the initial poor rescue action might result in worse outcomes. We need deeply investigation to answer this question. Secondly, the healthy swine model is not always indicated a real condition of patients in clinical setting. People in VF usually suffered from coronary artery occlusion or asphyxia; besides, the successful resuscitation are not usually benefited from only CPR and counter shock if suspected coronary artery was not under revascularization. After all, despite these limitations existed, the facts that the suboptimal CPR impaired CPP was confirmed, and if it occurs, even a delayed optimal CPR may fail to improve the limited survival opportunities.

## 5. Conclusion

In this porcine model of prolonged cardiac arrest, even four minutes of initial poor quality of CPR compromises the survival outcome.

## Figures and Tables

**Table 1:** Baseline characteristics.

	G-CPR (*n* = 7)	P-CPR (*n* = 7)	*P* value
Body weight (kg)	31.64 ± 2.37	31.93 ± 2.42	*0.82 *
Thoracic A-P diameter (cm)	22.27 ± 0.56	22.07 ± 0.73	*0.58 *
Hemodynamic status			
Mean aorta pressure (mmHg)	103.86 ± 21.67	105.14 ± 14.01	*0.90 *
Right atrium pressure (mmHg)	1.24 ± 0.77	0.93 ± 0.67	*0.43 *
Heart rate (bpm)	112 ± 12.70	114.29 ± 13.94	*0.75 *
Cardiac output (L/min)	4.76 ± 0.72	4.49 ± 1.19	*0.76 *
Blood-gas analysis			
Core temperature (°C)	37.90 ± 0.42	38.10 ± 0.58	*0.47 *
pH	7.49 ± 0.10	7.54 ± 0.13	*0.39 *
PaCO_2 _(mmHg)	36.61 ± 1.85	36.36 ± 1.26	*0.77 *
PaO_2 _(mmHg)	81.29 ± 8.54	83.00 ± 9.49	*0.73 *
Lactate (mmol/L)	1.76 ± 0.30	1.86 ± 0.34	*0.57 *

Based on analysis of variance test as appropriate. Values are expressed as mean ± SD.
